# Biomarkers of Postpartum Depression: A Narrative Review

**DOI:** 10.3390/jcm12206519

**Published:** 2023-10-14

**Authors:** Stefan Modzelewski, Aleksandra Oracz, Kamila Iłendo, Aleksandra Sokół, Napoleon Waszkiewicz

**Affiliations:** Department of Psychiatry, Medical University of Bialystok, pl. Wołodyjowskiego 2, 15-272 Białystok, Poland; aleksandra.julia.oracz@gmail.com (A.O.); kamila.ilendo@gmail.com (K.I.); ola.sokol24@gmail.com (A.S.); napoleon.waszkiewicz@umb.edu.pl (N.W.)

**Keywords:** postpartum depression, marker, biomarkers, peripartum, postpartum, cytokines, HPA, oxidative stress, genetics, nutrients, pathogenesis

## Abstract

Postpartum depression (PPD) is a disorder that impairs the formation of the relationship between mother and child, and reduces the quality of life for affected women to a functionally significant degree. Studying markers associated with PPD can help in early detection, prevention, or monitoring treatment. The purpose of this paper is to review biomarkers linked to PPD and to present selected theories on the pathogenesis of the disease based on data from biomarker studies. The complex etiology of the disorder reduces the specificity and sensitivity of markers, but they remain a valuable source of information to help clinicians. The biggest challenge of the future will be to translate high-tech methods for detecting markers associated with postpartum depression into more readily available and less costly ones. Population-based studies are needed to test the utility of potential PPD markers.

## 1. Introduction

Postpartum Depression (PPD) is a disorder that negatively affects not only women’s mental health, but also the relationship between mother and child itself. According to the DSM V classification, postpartum depression has its onset in the perinatal period. In terms of the ICD-11, PPD encompasses both pregnancy and the few months after delivery. Detecting patients with an increased risk of the disease would allow the implementation of appropriate treatment, which in turn would help in the prevention of potential complications.

Assaying biomarkers from biological material in bodily fluids can help in a minimally invasive way to assess the risk of the disorder, confirm the diagnosis and prognosis, and monitor the treatment of depression. The markers can provide insight into the etiopathogenesis of a depressive disorder, supporting the various theories of disease pathogenesis by providing indirect evidence. In our review, we have collected studies that significantly impact the current understanding of PPD and remain a valuable source of knowledge about the biomarkers of the described disorder.

## 2. Methods

PubMed and Web of Science databases were searched using the following key words: “postpartum depression”, “biomarker”, “marker”, “etiopathogenesis”, “neuroendocrine”, “inflammation”, “inflammatory”, “cytokines”, “kynurenine”, “oxidative stress”, “genetic”, “HPA axis”, “vitamins”, “metabolic”, “lipid”, as well as combinations of these terms. We have included relevant articles in order to assess the various markers of depression as accurately as possible.

## 3. Immunological Markers: Relevance to PPD

**Scheme 1:**
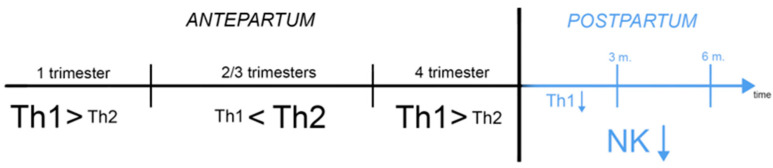
The immune response in the antenatal and postnatal period. Explanations in the text.

The etiopathogenesis of PPD is complex and multidirectional. As in MDD, PPD has a multicomponent pattern, in which inflammatory, hormonal, and genetic factors are attributed special roles [1]. Numerous studies point to the dysregulation of the immune system during pregnancy and childbirth. According to current medical knowledge, MDD patients have more frequently detected levels of inflammatory cytokines (e.g., IFN-γ, TNF-α) and an elevated Th1/Th2 ratio [2,3]. Pregnancies are characterized by similar data as a result of the immune switch (activation of Th1-related cytokines, see below) that occurs in the mother’s body before delivery [4]. During pregnancy, NK cells (Uterine Natural Killers, producing—among other cytokines—IFN-γ) significantly impact the production of pro-inflammatory cytokines (Scheme 1) [5].

Moreover, they remain in suppression after delivery until about 6 months [6], which correlates with reports that NK activity is reduced in depression [4].

Studies indicate that about 60% of kynurenic acid [7] in the CNS is of peripheral origin and enters through the blood–brain barrier (BBB).

**Figure 1:**
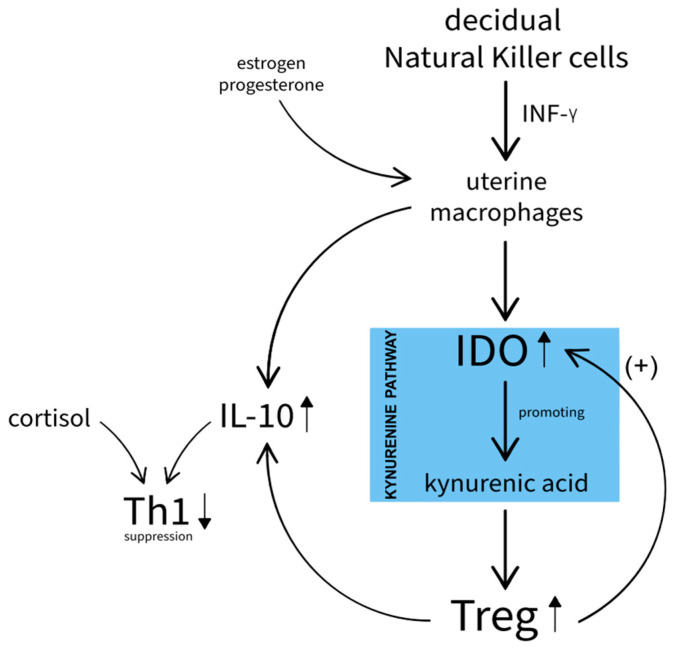
IDO-related transformations in the placenta.

Pregnancy, while remaining a special period for the functioning of the immune system, is associated with high enzyme activity of the kynurenine pathway, whose products are harmful to cells of the central nervous system (CNS):

The temporal macrophages (activated by IFN-γ produced by uterine NK) produce indoleamine 2,3-dioxygenase (IDO), an enzyme that is essential for inhibiting the cytotoxic response to the developing embryo (Figure 1). 

As a result, kynurenic acid is produced to promote the differentiation of Regulatory T cells (Treg) [8,9]—which also enhance IDO expression—on dendritic cells. After delivery, there is a decrease in placental melatonin, a physiological inhibitor of the kynurenine pathway [10]. Melatonin is now known to have anti-inflammatory effects that may also translate to the CNS [11].

Animal studies suggest that products of the kynurenine pathway are also formed in response to lipopolysaccharide (LPS) [12]. However, it is worth noting that the action of kynurenine pathway enzymes and products in the CNS is more complex and probably not as beneficial as in the placenta and embryo. Astrocytes and microglia convert the peripherally delivered kynurenic acid in the CNS to products of the kynurenine pathway including cytotoxic quinolinic acid (QUIN) [7,13]. In addition, pro-inflammatory cytokines as well as cortisol promote TDO and IDO enzyme activity, which increase the availability of tryptophan for the kynurenine pathway, thereby decreasing serotonin production [14]. Delivery is associated with a sharp increase in glucocorticosteroids (GCS), which inhibits the immune response, but also paradoxically exhibits the opposite effect by increasing the expression of receptors for cytokines [15,16]. Additionally, macrophages, probably the most essential immune cells of pregnancy, possess receptors for steroid-like hormones. Thus, they may themselves produce hormones of the HPA axis (hypothalamic-pituitary-adrenal axis). It is, however, believed that in the case of these cells, those substances have no peripheral, but only auto and paracrine action. Pregnancy, also seen as an immunologically complex process supporting the implantation of the embryo and then protecting it from the cytotoxicity of the immune system, is often described as a period of initial predominance of Th1 over Th2 cytokines [17], with a subsequent switch in favor of Th2, recognizing the huge role of macrophages, which promote transformation during its course. These immune dysregulations share features with the inflammatory theory of depression, in which a disturbed ratio between Th1/Th2 cells is also observed [18]. The period of childbirth itself, associated with tremendous stress on the body, mobilization of the HPA axis, and increased inflammatory response [1], can be considered a causal factor in depression. The initial Th1 suppression of the postpartum period is consistent with reports typical of MDD [2].

The exacerbation of *rheumatoid arthritis*, a Th1-dependent disease, about 3 months after delivery, indicates a long recovery period for lymphocyte subpopulations [19]. In addition, the role of inflammation and the immune system as central to postpartum mood is underscored by the drug brexanolone (an analog of allopregnanolone, which in turn is a metabolite of progesterone), a modulator of the GABA-a receptor, which is thought to inhibit the inflammatory response elicited by Toll-like receptors 4 and 7 and the activity of kynurenine pathway enzymes [20,21]. It is noteworthy that estrogen and progesterone levels drop shortly after childbirth [22]. These hormones are able to modulate the macrophage response via the NF-κB pathway in favor of Th2 [23], which explains their levels in the later trimesters of pregnancy, i.e., when the anti-inflammatory response begins to dominate. Brexanolone inhibits the LPS-induced response and reduces the levels of pro-inflammatory cytokines. Some of these, such as IL-1β, and IFN-γ in the CNS, have a negative effect on the neurogenesis of brain structures, such as the hippocampus, by affecting BDNF (brain-derived neurotrophic factor) [24]. GABA-ergic conductance, on the other hand, appears to have a positive effect on its neuromodulation. These interactions underscore the importance of the centrally mediated process. The stress of childbirth may also interfere with neuromodulation and reduce the plasticity of synapses in the hippocampus, especially since high concentrations of GCS inhibit hippocampal neuromodulation, presumably via the NMDA receptor [25].

Peripherally produced cytokines themselves can also have an effect, penetrating the BBB barrier to the CNS by inducing cyclooxygenase-induced (COX-2) prostaglandin-2 (PGE2) nitric oxide (NO) activity, as well as stimulating perivascular macrophages, microglia, and astrocytes to produce cytokines, or, as mentioned above, reducing BDNF levels and activating the IDO enzyme to enhance excitotoxicity [14].

### 3.1. Kynurenine Pathway

During pregnancy, kynurenine pathway enzymes are highly expressed in the placenta, and their activity is crucial for regulating the maternal immune response to the fetus. It is suggested that the tryptophan-degrading enzyme indoleamine-2,3-dioxygenase (IDO) is activated by inflammatory factors—including cytokines produced by Th1 and NK lymphocytes. As a result of activation of the so-called TRYCATs pathway (tryptophan catabolite pathway), instead of serotonin, kynurenine is produced—a neurotoxic compound that increases the risk of neurodegenerative processes. The decreasing amount of available serotonin leads to depressive disorders [18].

The activity of IDO and other enzymes of the pathway increases with placental development. Some of the pathway’s metabolites are neuroactive, including kynurenic acid (KYNA) and quinolinic acid (QUIN), and affect glutamate neurotransmission. Studies show that kynurenine and quinolinic acid assayed in the 2nd trimester of pregnancy are good predictors of PDD severity, progression, and risk [26,27,28].

### 3.2. Hormone Markers: Relevance to PPD

Sex hormone levels are significantly increased during pregnancy (estrogen increases 50-fold from the follicular phase and progesterone increases 10-fold) [29]. Their reduction after delivery may be a significant stressor and play a role in the mechanism of depression induction [22]. Smith 1991 pointed to estrogen as a factor in macrophage activation enhancing macrophage action and phagocytosis [30], which would be consistent with inhibiting the activation of more complex immune mechanisms during pregnancy that could put the developing fetus at risk. 17-beta-estradiol modulates glutamatergic conduction in the hippocampus, leading to increased synaptic excitability in the long term [31].

**Figure 2:**
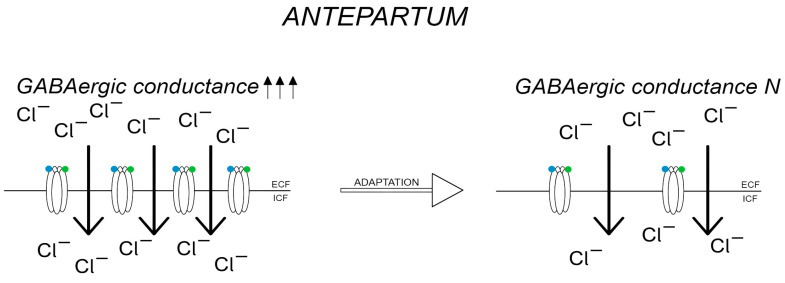

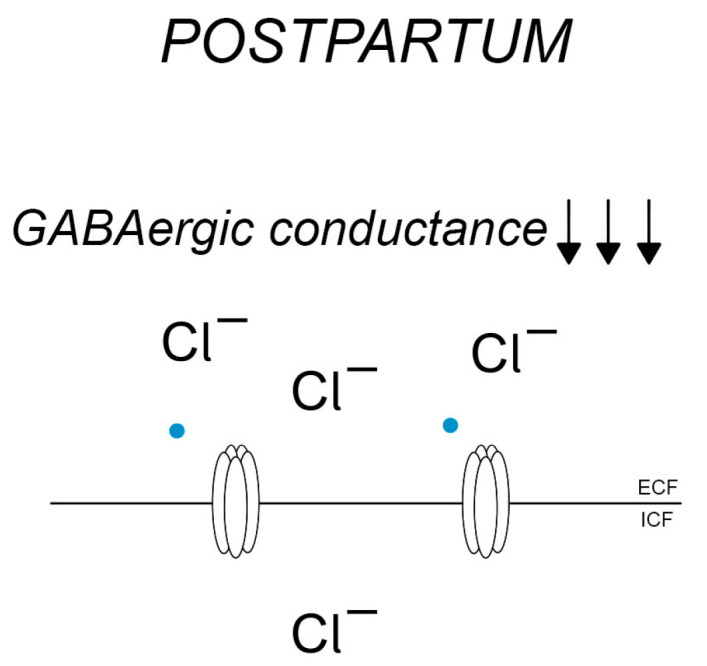
Hypothesized role of the GABA receptor in PPD. During pregnancy, there is an increase in allopregnanolone and an enhancement of GABA conductance. In response, GABA receptor down-regulation occurs. In the postnatal period, there is an initial attenuation of GABA conductance as a result of low receptor abundance and decreased allopregnanolone concentrations. 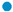
—GABA, 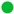
—allopregnanolone.

This situation may exacerbate the cytotoxic effects of glutamate, which could lead to the changes observed in depression. On the other hand, progesterone, through its active metabolite, allopregnanolone, promotes GABAergic conduction, which conditions adequate excitability of the hippocampus through its tonic inhibition [32]. Allopregnanolone is an allosteric modulator of the GABA-a receptor, whose levels are reduced in animal models during pregnancy; in turn, a sharp drop in progesterone levels leads to an increase in the alpha and epsilon subunits of the GABA-a receptor [33]. An attempt to explain this phenomenon was made by Maguire et al. According to them, the decrease in GABA receptor expression may be a physiological response of the organism to avoid excessive inhibition in the CNS (Figure 2).

On the other hand, after pregnancy, when progesterone concentrations return to normal, there would be an increase in GABA-a receptor expression to maintain constant GABAergic inhibition [34]. As a result of exposure to stressors, this phenomenon could lead to symptoms of depression—for example, due to incomplete resynthesis of GABA receptor subunits.

Progesterone may also produce widespread effects on other hormones through GABA conductance. The GABA-a receptor is also crucial in inhibiting Corticotropin-Releasing Hormone (CRH) production in the PVN (paraventricular nucleus) [35], suggesting that progesterone is protective against excessive stress, while the effects of GABA conductance on prolactin and oxytocin may be crucial in preventing preterm labor [32].

Estrogen promotes aerobic metabolism in the brain vasculature and increases the production of cytochrome C and manganese superoxide dismutase, leading to a decrease in the production of reactive oxygen species [36]. The hormone has also been linked to impaired serotonergic transmission, and its impact on u-opioids may inhibit GABA-b in the hypothalamus [31]. It also promotes the formation of BDNF and VEGF, which can constrain inflammation [37]. In a study on a group of women with a history of PPD, the introduction of hormone doses at pregnancy-like level and their decrease mimicking the transition to the postpartum period resulted in the appearance of depressive symptoms [38,39], suggesting that there is a subgroup of women who are sensitive to hormonal fluctuations.

**Figure 3:**
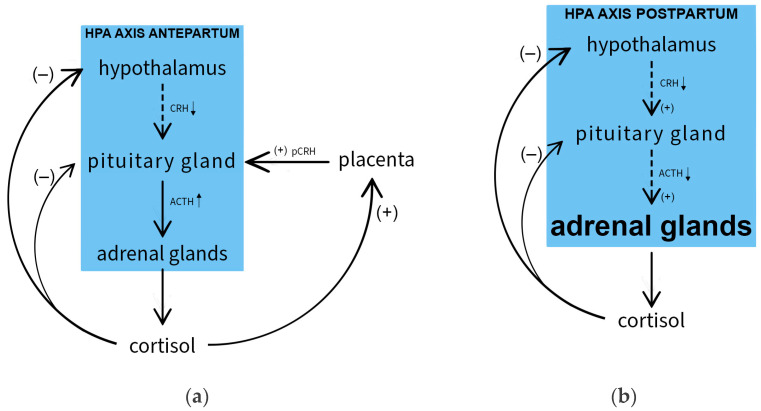
HPA axis during pregnancy AND postnatally: (**a**) stimulation of cortisol and ACTH secretion in the presence of pCRH; (**b**) in the postnatal period, there is no longer an additional source of CRH. The HPA axis is in suppression and slowly returns to physiological functioning.

It is not only sex hormones, but also the HPA axis during pregnancy and childbirth that may be involved in the etiopathogenesis of depression. Adrenal suppression shortly after childbirth is comparable to the levels found in patients suffering from Cushing’s disease and depression [40]. This is due to placental production of CRH (pCRH), which leads to ACTH stimulation and adrenal hypertrophy during pregnancy [41]. During labor and after delivery due to the removal of the placenta, the HPA axis is exposed to severe changes owing to an acute decrease in placental CRH levels. After delivery, significant decreases in CRH and ACTH concentrations are observed compared to the 3rd trimester. Cortisol, on the other hand, remains physiologically normal, which is likely related to induced adrenal hypertrophy during pregnancy (Figure 3).

Rapid changes in CRH concentrations as well as chronic hypercortisolism during pregnancy can be considered potential stressors. Hypercortisolism and the epinephrine-stimulating effects of CRH may in the long run contribute to the exacerbation of chronic inflammatory processes [42,43]. Studies of animals subjected to chronic stress show reduced survival of hippocampal cells [44]. However, removal of the adrenal glands did not result in improved survival of those cells [45,46]. This suggests a more complex mechanism of action of GCS, possibly mediated by the NMDA receptor [47]. Elevated levels of Interleukin 1β (IL-1β) and reduced levels of BDNF were observed in rat hippocampal biopsies. IL-1β has shown an important role in blocking the suppressive effect of GCS on lymphocyte proliferation, and administration of its antagonist abolished the exacerbation of the stress response in rats [12,43].

## 4. Multiple Pregnancy and Subsequent Pregnancies

A separate issue beyond the scope of our review concerns the risk of recurrence of PPD in subsequent pregnancies. The immune switch, constituting a period of temporarily heightened cellular response, is meant to induce labor [48]. That moment can lead to increased fetal tissue expression and the formation of cytotoxic T lymphocytes. Most cells, as a result of postnatal depletion, remain in suppression [6]. However, the example of at least Rh disease points to the importance of memory cells. Kinder et. al. shows the roles of CD8+ T cells and the system that inhibits their toxicity on fetal cells [49]. Interestingly, fetal FOXP3+ alloreactive CD4+ regulatory T cells retain after primary pregnancy (mouse study). There is also a link between their accelerated re-development after fetal antigen stimulation and protection against the death of the fetus in a secondary pregnancy [50]. The Blochs study (mentioned below) [51] indicates that the most significant role for the subject’s mood seems to lie in hormonal fluctuations in a subgroup of women sensitive to their changes. However, a theoretically difficult delivery during the first pregnancy could lead to complications in maintaining the next pregnancy and immune imbalances, thus indirectly increasing the risk of PPD.

Multiple childbirths are also considered a risk factor for PPD [52].

According to a study by Choi et al., depression symptoms were measured 9 months after delivery using a shortened version of the Center for Epidemiologic Studies Depression Scale. The results were surprising. They found that mothers who gave birth multiple times were 43% more likely to have moderate/advanced symptoms of depression 9 months after delivery, compared to women who gave birth once [53].

There is also a belief that multiple births are an indirect risk factor for PPD, by affecting: low birth weight of the baby, gestational hypertension, as well as the initiation of breastfeeding and the method of feeding [54]. 

The baby’s low birth weight and premature delivery make it more susceptible for infections, putting the mother under stress, which can result in PPD [55]. 

In addition, the infants who were breastfed within 2 h of birth were more likely to reach a lower weight at 4 weeks than those who were breastfed more than 2 h after birth. This increased the risk of postpartum depression in mothers [56,57]. 

## 5. Review of Hormonal Markers

### 5.1. Estrogen and Progesterone, Allopreganolone

During the third trimester of pregnancy, estrogen levels increase 50-fold, while progesterone reaches a 10-times-higher concentration compared to the maximum levels in the monthly cycle. After childbirth, there is a significant decrease in the concentration of hormones, which from a high peak decrease to the lower limits of the physiological norm. During pregnancy, those hormones are essential for the normal development of the fetus, performing protective functions—suppressing the immune response to the developing embryo and also participating in the proliferation of hippocampal cells [58]. Special attention should be paid to progesterone, the derivative allopregnanolone of which has been shown to be effective in the treatment of PPD [59]. This underscores the vital importance of sex hormones, and provides an argument for the assumption that PPD is the result of rapid decreases in hormones (mainly progesterone and its metabolites) after childbirth [60,61], leading to changes in the modulation of GABAergic conduction in the CNS [61]. The role of sex hormones in the disorder is described in more detail in the section above. 

The study by O’Hara et al. showed lower serum estradiol levels measured in women with PPD compared to healthy women, at 36 weeks pregnant and day 2 postpartum [62]. In contrast, no changes were observed in progesterone levels between the groups. Another study that measured salivary progesterone also showed no significant differences between the studied groups [63]. However, another trial indicated elevated progesterone levels (serum) on day 7 postpartum in a group of affected women [22,64].

Other studies that measured hormone levels in the blood indicate [65] that the greater the drop in progesterone, the higher the mood deterioration, which correlates with the GABAergic theory in PPD. However, this study was on a small group of subjects, and the postpartum women’s follow-up lasted only until week 10. In contrast, a study by Harris et al. found no differences in progesterone (measured in blood and saliva) and estrogen levels measured in blood at 6–8 weeks postpartum between women with PPD and healthy women [66]. However, salivary progesterone and prolactin were positively correlated with depression if mothers bottle-fed rather than breastfed. The duration of the study also remains short, making it difficult to draw firm conclusions.

Data collected by Zonana et al. [67] indicate that the low levels of estrogen observed after delivery may be associated with PPD, suggesting an important role of steroid hormones especially in the first days after delivery and their significance as a possible trigger for depression. In contrast, in a study by Pearson et al. [68], levels of progesterone metabolites were significantly higher during pregnancy in depressed women, supporting the theory outlined by Maguire et al. According to that theory, there is a reduction in neuronal sensitivity to allopregnanolone during pregnancy as a result of high concentrations of this neurosteroid. Given the pathophysiology of the disorder, allopregnanolone concentrations in the postpartum period may be an important diagnostic marker and determinant of response to treatment [34].

### 5.2. HPA Axis

The period of pregnancy and childbirth is subject to dysregulations of the HPA axis. In particular, there are large fluctuations in pCRH (placental CRH), which is secreted by the placenta and does not respond negatively in feedback to cortisol concentrations. This causes its levels during pregnancy to be up to 30 times [69] above the norm. ACTH, due to free CRH, reaches its highest values (5 times the norm) in the 3rd trimester, while cortisol increases 2–3 times [22].

Placental CRH plays an important role in modulating the immune response to the developing fetus, while cortisol, through the nuclear factor kappa B (NF-κB) pathway, promotes further CRH secretion in a positive feedback mechanism. This pathway is stimulated by various stressors and can potentially lead to preterm labor as a result of excess pCRH [70]. The increase in cortisol-binding proteins stimulated by estrogen and adrenal hyperplasia results in normal levels of cortisol despite the postpartum observed rapid decline in ACTH and pCRH, with concomitant weakening of the HPA axis until about 12 weeks postpartum. Moreover, these phenomena make pregnancy and the first days postpartum a period of impaired response to the DMT (Dexamethasone) suppression test, bridging previous studies of the suppression test as a marker of non-pregnancy-related depression [18,71].

The entire axis is at a risk of a sharp decline due to the loss of pCRH, which may be considered one of the risk factors for postpartum depression. A study by Bloch et al. also indicated that a decrease in estrogen and progesterone in women with a history of PPD causes a significant increase in cortisol detected in saliva, blood, and urine [22]. However, most studies have not found a correlation between cortisol levels and PPD [51]. Yim et al. indicated that pCRH may be a significant marker of PPD [72], but later studies revealed conflicting findings [73]. Despite the unpromising results, a study of rats proved that it is possible to induce depressive symptoms and reduce promatogenic behavior in the absence of the ability to inhibit the HPA axis, providing evidence for the importance of the HPA axis in postpartum depressive disorder [74]. On top of this, a metabolite of pregnanolone, allopregnanolone, while exhibiting effects on GABA conductance, inhibits the mRNA expression of CRF, a major activator of the HPA axis in response to stress, leading to a decrease in blood concentrations of ACTH and cortisol, which represents one of the brexanolone’s mechanisms of action.

It is important to note that PPD can be characterized by different subtypes of depression, which will be reflected in HPA performance. Cortisol levels during pregnancy are similar to the ones typically found in melancholic depression, while the postpartum period may be associated (due to HPA inhibition) with atypical depression [75,76]. Moreover, recent literature data for the atypical subtype indicate that women suffering from mood disorders in the perinatal period had significantly reduced morning cortisol [77]. This observation makes it possible to consider cortisol levels measured in the morning as an endophenotypic and diagnostic biomarker.

### 5.3. Prolactin and Oxytocin

The role of hyperprolactinemia in PDD remains controversial. It is thoroughly discussed in a review by Szpunar et Parry [77]. Prolactin, stimulated by estrogen, may exceed the norm as much as 50 times during pregnancy. Its levels normalize by 3 weeks postpartum in non-breastfeeding women and much later, within months, in lactating women. To date, studies of blood prolactin levels are insufficient to draw firm conclusions. However, it is important to note the reports indicating that women suffering from PPD or with a prior history of depression have more difficulty with breastfeeding [64,78]. Some studies also show lower prolactin levels in these women [79]. Prolactin should be assayed over many weeks as single measurements may not have high diagnostic value. There are no consistent conclusions from the studies conducted to date, which may be due to differences in the timing of sampling. However, it should be remembered that prolactin is not a hormone secreted in a diurnal rhythm, which further complicates the matching of the results of different studies [80]. Okun et al., conducting a study on 56 women at high risk of developing MD, collected samples for 17 weeks after delivery. His study found no correlation between PPD and prolactin levels [81].

Oxytocin, which is associated with the development of maternal care and breastfeeding, is often associated with symptoms of PPD, such as decreased interest in the baby and feeding problems. In Deems and Leuner’s review, the studies collected did not give a clear conclusion on the relevance of oxytocin in PPD. However, much of the presented work indicates that oxytocin could be a useful marker of the PPD risk [79]. A study by Jobst et al. revealed that in mothers with PPD symptoms, oxytocin decreases from 38 weeks of gestation to day 2 postpartum against a continuous increase in healthy mothers [82]. Massey et al. attributes the inconsistent results described in the above-mentioned review to different study methods or too small groups of patients included in the studies [83].

### 5.4. Thyroid Hormones

In the population of patients with depression, thyroid hormone disorders are common [77]. Thyroid hormones can affect the mood, yet in the context of PPD, there is a lack of current data defining their importance as a biomarker. Both T3 and T4 blood concentrations increase during pregnancy, reaching their highest levels perinatally. However, as they increase, TBG (thyroxine-binding protein) levels also rise due to increased estrogen [84], keeping free hormone concentrations within normal limits [85]. The impact of placenta-secreted hCG, which can exert TSH-like effects, may also be relevant [86]. Nonetheless, current data do not allow linking TSH to PPD [77]. Bloch et al., who compiled data from the literature describing thyroid disorders in the context of PPD, highlight microsomal antibodies, against peroxidase and against thyroglobulin, which may be useful markers indicating the risk of developing thyroid dysfunction and developing PPD. However, the correlation between the two was low [22].

### 5.5. Conclusions

Given the high fluctuations of estrogens and progesterone combined with reports indicating the existence of a subgroup of women sensitive to hormonal fluctuations, estrogens and allopregnanolone may have the greatest value as potential biomarkers. Their oscillations, comparing concentrations in the 3rd, 4th trimester in addition to postpartum period, could be a predictor of the severity of the disorder as well as a prognostic marker (Table 1). The role of allopregnanolone is further emphasized by studies on the drug brexanolone. Considering that the central concentration of allopregnanolone appears to be particularly important, saliva levels of this marker should, in addition to ones in peripheral blood, also be considered. Cortisol, due to its unique concentrations during the postpartum period, is worth monitoring in order to characterize the type of depression—atypical versus melancholic.

## 6. Review of Inflammatory Markers

### 6.1. Tumor Necrosis Factor α (TNF-α)

TNF-α is a Th1-type multifunctional cytokine produced by macrophages. It is one of the most important inflammatory cytokines. In pregnancy, TNF-α affects hormone synthesis, placental architecture, and embryo development. In addition, increased levels of TNF-α alter the secretion of immunomodulatory factors by the placenta, which in turn has implications for maternal immune cell function [87].

The role of TNF-α in the development of PPD is a contentious issue on which there are many conflicting scientific reports. For example, Corwin et al. found a positive correlation between PPD and levels of circulating interleukin IL-6 and tumor necrosis factor TNF-α at around the 15th week of pregnancy[88]. In contrast, a study by Buglione-Corbett et al. demonstrated that elevated TNF-α serum levels in patients who were in their 3rd trimester of pregnancy translated into a reduction in the total score on the Edinburgh Postpartum Depression Scale (EPDS) [89].

Simpson et al. argued that IL-6, IL-10, and TNF-α levels did not change significantly from the 3rd trimester to 12 weeks postpartum, but found an association between IL-6 and IL-10 levels in late pregnancy and PPD symptoms [90]. In another study conducted at the 24th week of pregnancy, IL-6 and TNF-α levels were not associated with prenatal symptoms [91].

Meta-analyses on biomarkers of depression in nonpregnant individuals repeatedly highlight the importance of TNF-α as a diagnostic marker as well as a marker of treatment response [18].

### 6.2. C Reactive Protein

C Reactive Protein (CRP) is classified as one of the so-called acute phase proteins. It participates in the immune response by facilitating complement binding. During physiological pregnancy, median CRP values reach higher levels than standardized values for nonpregnant women and remain at increased levels even during delivery [92].

There are a number of conflicting scientific reports on the importance of CRP as a marker of PPD. 

A study by Liu et al. including 296 women, 45 of whom showed symptoms of PPD, found that High-Sensitivity CRP (Hs-CRP) serum levels in women with PPD were significantly higher than in women not suffering from PPD. This indicates a strong association between elevated Hs-CRP serum levels on admission and the development of PPD within 6 months [93]. In contrast, Miller et al. found no association between CRP serum levels and PPD symptoms [94]. 

Notably, the study of the relationship between CRP levels and the development of PPD is hampered by the need to interpret the results after considering the patient’s BMI (a higher BMI before pregnancy was associated with increased CRP levels over time), the anti-inflammatory medications used, and personal genetic variants [94].

It is worth mentioning that literature identifies CRP protein as an important non-specific immune marker of MDD. It is noted for its wide use as not only a diagnostic and predictive marker, but also as a marker of treatment efficacy [18].

### 6.3. Transforming Growth Factor Beta (TGF-Beta β)

TGF-β is a family of cytokines that play an important role in both the initiation of inflammation, its extinction (including the T-cell response), but also in repair processes, i.e., fibrosis and angiogenesis [95,96].

During pregnancy, TGF-β is present in amniotic fluid and the placenta. It has been suggested that TGF-β1 protects the fetus from potentially harmful excessive activation of pro-inflammatory cytokines by the mother’s body [97].

Studies have observed increased levels of TGF-β2 and decreased levels of TGF-β1 and TGF-β3 in the colostrum of mothers with PDD [98].

Further studies are needed to demonstrate the usefulness of TGF-β as a diagnostic marker for PDD.

### 6.4. IL-10

IL-10 is one of the anti-inflammatory interleukins produced by T lymphocytes, B lymphocytes, and macrophages. Its reduced levels contribute to dysregulation of pro-inflammatory cytokine levels, which in turn, according to the cytokine theory of depression, is associated with the occurrence of depressive disorders [99].

Studies demonstrate that IL-10, assayed in the blood, can be a peripheral predictive marker of postpartum depression symptoms [90]. Decreased levels of IL-10 in the third trimester of pregnancy are an indicator of the severity of depressive symptoms both during pregnancy and after delivery [100]. It has been suggested that a decrease in IL-10 levels during the third trimester of pregnancy may be indicative of decreased Th2 lymphocyte activity, which in turn is thought to influence the severity of depressive symptoms even before delivery [90].

IL-10 is also a predictive marker of severe depressive disorders. Nonpregnant patients with MDD have increased levels of this interleukin compared to healthy individuals [101].

### 6.5. IL-18 

IL-18 belongs to the IL-1 group of pro-inflammatory cytokines. It is produced as an inactive precursor, after which it is activated with the participation of caspase 1. It differs from the other cytokines in its family by being present in almost all the cells of a healthy organism. It plays an important role in the production of IFN-γ from T lymphocytes and NK cells [102].

It has been suggested that IL-18 may play a key role in regulating the Th-dependent response of the mother’s body during early pregnancy [103,104]. 

Higher levels of IL-18 are observed in the plasma of women suffering from depression during pregnancy than in pregnant women without symptoms of the disease [105].

Some studies conducted in mothers with PPD symptoms reveal a positive correlation between depressive symptoms and maternal IL-8 levels, as well as IL-18 levels in the umbilical cord [106].

Further research is needed if only to more precisely assess the role of IL-18 in pregnancy and its relationship to postpartum depression, especially since elevated levels of IL-18 are also observed in individuals with depressive disorders unrelated to pregnancy [107].

### 6.6. Chemokines 

Chemokines play a key role in the early stages of pregnancy: not only in the recruitment and functional regulation of the immune cells of the endometrium, but also in blastocyst/embryo implantation into the uterus and trophoblast invasion [108].

Fractalkine/CX3CL1 is a membrane-bound or soluble chemokine found in neurons. It inhibits serotonergic neurotransmission by enhancing the action of GABA on serotonergic neurons. In addition to its angiogenic VEGF-A effects, it also has neurotrophic and neuroprotective functions in the central and peripheral nervous systems, which may be relevant to the development of depression [109].

Elevated levels of chemokines, i.e., CCL2, CCL11, CCL5, and CXCL10, correlate with active and psychosomatic symptoms of the menstrual cycle, while menstrual symptoms including pain, cramps, and gastrointestinal complaints may be related to the action of CCL5 [110].

Interleukin-8 (IL-8), part of the CXC chemokine family known as CXCL8, regulates pathological angiogenesis and tumor growth. It is also responsible for the occurrence of metastasis. IL-8 and its receptors CXCR1 and CXCR2 have been observed on endothelial cells and proven to play a role in their proliferation. IL-8 is one of the main chemoattractants for neutrophils; moreover, it can activate these cells [111].

A study by Edvinsson et al. showed reduced levels of fractalkine (CX3CL1) in a group of patients suffering from depression during pregnancy [109].

In another study (Petralia et al.), PPD patients were tested for CCL2 chemokine receptor (CCR2) and CCL5 chemokine levels, among others. It was documented that blood levels of CCR2 were significantly lower compared to the control sample (comparable values to MDD patients), while CCL5 levels were significantly higher in PPD patients [112].

In the Camacho-Arroyo et al. study, high levels of chemokines CCL4 and CCL11 correlated with the patients’ HDRS scores [112].

Maes et al. found that CXCL8 (IL-8) levels were associated with the severity of the condition. Elevated CXCL8 levels were also noted in the Corwin et al. review [88]. The researchers concluded that a high IL-8/IL-10 ratio may be a good predictor of PPD.

In the broader context of major depressive disorder (MDD), meta-analyses show that depressed patients have higher CCL2 chemokine levels compared to the control sample [113].

### 6.7. IL-1β

IL-1β is one of the major pro-inflammatory cytokines and, along with TNF-α and several other cytokines, is thought to be responsible for the patient’s characteristic behavior [114].

Corwin et al. found that pregnant women who reported depressive symptoms on day 28 after delivery also had elevated levels of IL-1β in a urine sample taken two weeks earlier [115].

Sha et al. demonstrated that increased levels of IL-1β in the blood of pregnant patients were associated with a worsening of depressive symptoms and an increased risk of severe disease [26].

Increased levels of IL-1β in depression are still a controversial issue. Some meta-analyses confirm such associations, while others show no such relationship [116].

IL-1 may also be a promising potential prognostic biomarker, as elevated levels of IL-1β mRNA in the blood may correlate with poorer response to the antidepressant treatment [117].

The underlying physiological mechanism for the link between early elevated IL-1β levels in the postpartum period and the onset of depressive mood several weeks afterwards is not yet fully understood. However, it is known that pro-inflammatory cytokines have the ability to stimulate the HPA axis, leading to increased cortisol secretion [118]. It is possible that in women with significantly elevated IL-1β levels, cortisol concentrations may also reach abnormally high levels, resulting in dysregulation of the HPA axis [119].

### 6.8. IFN-γ

IFN-γ is secreted by Th1 lymphocytes as well as NK cells. This cytokine is part of the inflammatory response and has been found at high levels in the precentral frontal cortex of female rats with induced depression at the end of pregnancy, indicating that it is important for the functioning of the neuroimmune system in the context of PPD [120].

During pregnancy in women, IFN-γ levels are higher in the 1st trimester, promoting angiogenesis and embryo implantation [121]. Because NK cells remain in suppression until 6 months after pregnancy [6], it is secreted mainly by Th1 in the postpartum period. Study data indicate that Th1 lymphocytes remain in suppression until 3 months after birthgiving [19]. Results indicate that an increase in IFN-γ correlates with the return of Th1 lymphocyte activity [122]. In a study by Groer et Morgan, saliva samples were collected from mothers with PPD symptoms from 4 to 6 weeks after delivery, and reduced serum IFN-γ levels were observed compared to healthy controls [5]. This indicates increased Th1 suppression in women with PPD, compared to healthy mothers in the initial weeks after delivery [122].

### 6.9. IL-6 

IL-6 is a regulatory and pro-inflammatory cytokine that activates both monocytes and microglia, as well as other cell types of the innate immune system [27]. It is also the most studied, with higher levels associated with increased risk of depression, as confirmed in several meta-analyses [18].

Sha et al. achieved high accuracy in predicting the severity of depression by determining IL-6 in the plasma of patients in the 2nd trimester of pregnancy. This made it possible to determine the risk and estimate the severity of symptoms appearing as early as the 3rd trimester of pregnancy [26].

A study of women with affective disorders found that elevated blood levels of IL-6 were associated with a cellular immune response in patients who were assessed for suicide risk [26]. Increased levels of IL-6 in cerebrospinal fluid were also observed in individuals who had attempted suicide [27].

IL-6 may be related to the severity of stress, as evidenced by a study in rats where 9 different mild stressors were randomly applied for 12 weeks to maintain mild stress for a longer period of time. Wang et al. noted that microglia activity produced pro-inflammatory cytokines, including IL-6, IL-1β, and TNF-α. Expression of the pro-inflammatory mediator genes IL-1β, IL-18, and IL-6 was also detected in the hippocampus [123].

Nevertheless, a small number of studies have found no correlation between IL-6 levels and EPDS, depressive symptoms, or stress-related changes [18].

### 6.10. IL-2 and Soluble Interleukin-2 Receptor (sIL-2R)

IL-2 plays a key role in the immune response as a T-cell activator, inducing regulatory and effector T cells into their respective subtypes [27]. The action of IL-2 is mediated by the IL-2 receptor (IL-2R), present on the cell membranes of activated T cells.

Research results regarding the impact of IL-2 levels on PPD vary widely.

No significant differences in plasma IL-2 levels were usually observed [26]. However, Zhu et al. indicated that low plasma IL-2 levels also contributed to an increased risk of PPD [1].

A review by Carvalho et al. confirmed elevated blood levels of sIL-2R in patients with MDD [124].

### 6.11. IL-4 

IL-4 is an important anti-inflammatory factor that selectively activates M2 macrophages. Research results indicate that IL-4 plays an important role in cerebral neuroimmunomodulation [125].

IL-4 was found to have beneficial effects on cognitive function, which may be related to its ability to stimulate astrocytes to produce brain-derived neurotrophic factor or nerve growth factor [126].

Patients with bipolar disorder (BD) showed significantly higher levels of IL-4 than patients with MDD, implying that IL-4 may be a biomarker for differentiating MDD from BD [126].

A meta-analysis by Osimo et al. found that interleukin-4 (IL-4), one of the most important anti-inflammatory cytokines, is reduced in depression [18].

However, no link was found between IL-4 levels and an increased risk of PPD or postpartum anxiety (PPA) [127].

### 6.12. IL-8

IL-8 is a pro-inflammatory mediator known to stimulate peripheral mechanisms mediated by macrophages and granulocytes [27]. An increase in IL-8 secretion in the postpartum period has been reported. Elevated plasma levels of IL-8 were associated with a higher risk of PPD [128]. Levels of the pro-inflammatory cytokine IL-8 increase after estradiol and cortisol drop to low concentrations in the early postpartum period [27].

### 6.13. IL-17

IL-17A is a cytokine associated with chronic inflammation. It is secreted by T helper 17 (Th17) lymphocytes and plays a key role in the activation of the immune system and in the pathogenesis of some autoimmune diseases, such as multiple sclerosis [129].

No link was found between plasma IL-17A levels and the incidence of MDD in older adults. It has previously been shown that cognitive impairment in late-life depression is associated with significant abnormalities in immunoinflammatory pathways [130].

Min et al. documented a positive correlation between elevated serum IL-17 levels and an increased risk of PPD and PPA. It is possible that this is due to increased levels of Th17 cells, which may be involved in regulating brain inflammation by secreting pro-inflammatory cytokines such as IL-17A, which led to the development of PPD [127].

### 6.14. BDNF

Brain-derived neurotrophic factor (BDNF) acts in the brain primarily as a neuromodulator, playing an important role in the growth, differentiation, and maintenance of synaptic plasticity in the nervous system [131].

Gao et al. found that women with PPD had significantly lower plasma BDNF concentrations. Moreover, it was noted that patients whose symptoms disappeared after delivery had an increase in BDNF levels. BDNF levels were also found to be higher after childbirth than during pregnancy [132]. This may be related to fluctuations in cortisol and sex hormones.

The findings of the studies, however, are contradictory. Differences in sampling may be responsible for this (different results were obtained depending on whether plasma or serum was collected). BDNF is secreted by platelets into the serum during blood clotting; therefore, BDNF levels may be higher in serum samples obtained by centrifugation [133].

BDNF levels may be related to the suppressive effects induced by the stress associated with an episode of acute mental illness [134]. It was also found that women who had experienced stressful events in their past had lower serum BDNF levels on admission than women who had no such experiences [132].

### 6.15. IL-3

IL-3 is an important cytokine that has many biological functions in the immune response. Among other things, it is responsible for stimulation of cell growth, inhibition of apoptosis, and development and differentiation of all types of hematopoietic cells [135].

Metanalysis by Osimo et al. has shown that IL-3 blood levels are elevated in patients with depression [107].

### 6.16. Conclusions

Determining cytokines in the perinatal period remains quite difficult in relation to mood disorders (Table 2). Th1 suppression in the period up to 3 months postpartum means that determination of TNF-α and IFN-γ during this period will not yield reliable results. Childbirth as a stressor can physiologically lead to an elevation of IL-6 and IL-1β, and a decrease in BDNF and other cytokines shortly after or before labor, which reduces their diagnostic value. However, during gestation, they can provide information about the mother’s stress level. Low specificity and correlation with BMI limit the use of CRP. IL-10, an anti-inflammatory cytokine, can be a good marker for predicting the severity of a disorder, determination from peripheral blood, as its level can indicate how much immune variation will be involved in the delivery—and thus how severe the stressor itself will be. Paracrine changes around the placenta are particularly important for the survival of the pregnancy, whereas CCL-2 and fractalkine may be useful diagnostic markers of depression during pregnancy.

## 7. Nutrients

During pregnancy, nutritional requirements continually increase. The main reason for these changes is to sustain the metabolism and development of the mother’s reproductive tissues and the normal growth and development of the fetus.

Rupanagunta et al. noted that a considerable number of studies assessed the relationship between the diet and PPD from the perspective of dietary patterns and neurobehavioral influences. There is currently no statistically significant evidence to support an association between dietary nutrient intake and PPD [136]. 

### 7.1. Vitamin A

Consumption of vitamin-A-rich foods showed almost no association with depression among perinatal women, while in non-perinatal women consumption of such foods was associated with a lower likelihood of depression [137].

A significant reduction in serum levels of vitamin E and, to a lesser extent, vitamin A, has been noted in MDD patients [95].

### 7.2. B Vitamins

The B vitamin complex is involved in the metabolic processes of many body systems. Depending on its concentration, it is associated with anemia, or with neurological deficits, amnesia, and may also affect mental disorders [136].

Vitamin B6 is an essential cofactor of tryptophan metabolism and facilitates the conversion of tryptophan to the serotonin. For this reason, vitamin B6 can have a significant impact on reducing depressive symptoms [38].

Folic acid (vitamin B9; FA) also takes part in the biosynthesis of monoamine neurotransmitters (e.g., serotonin, dopamine, and norepinephrine) [138]. A review by Bodnar and Wisner has shown that low levels of folic acid can increase the risk of depression [139].

The results regarding the link between FA supplementation during pregnancy and the occurrence of PPD remain conflicting. FA supplementation during pregnancy has been identified as a protective factor against PPD in some studies, while others have failed to establish an association between FA supplementation and PPD [140,141,142].

Vitamin B12 is an essential micronutrient for neurological function. It is involved in regenerating methionine from homocysteine, which is a precursor to the biologically active molecule S-Adenosyl methionine (SAM) [143]. Levels of this vitamin are important for the baby’s birth weight and low levels can cause premature birth, acting as an additional stressor [144]. Women with suspected PPD had low plasma vitamin B12 levels [145].

### 7.3. Vitamin C

L-ascorbic acid, known as vitamin C, is a water-soluble antioxidant vitamin. The active form of vitamin C has a protective function by scavenging free radicals [146,147]. Ascorbic acid also acts indirectly as an antioxidant [148].

A review by Mortiz et al. described the neuroprotective impact of ascorbic acid, but such mechanisms are still poorly understood. Ascorbic acid supplementation has been shown to have antidepressant properties and improve the mood, probably due to its antioxidant effect [149]. Animal studies suggest that ascorbic acid supplementation influences serotonin concentrations in the striatum. It may also have a protective function against cortisol secreted in stressful situations [150,151]. Thus, it can be considered potentially useful in supporting the treatment of mood disorders, including depression [149].

### 7.4. Vitamin D

Vitamin D is a steroid molecule whose concentration in the body depends on the diet and ultraviolet radiation, which allows the synthesis of this vitamin in the epidermis [152]. One of the physiological functions of vitamin D is its key role in regulating calcium metabolism [153,154]. Amini et al. have suggested a link between vitamin D deficiency and symptoms of depression [155,156,157,158]. However, the authors of the systematic review note that few of these studies assessed the relationship between its insufficient levels in the body and the occurrence of PPD [155]. For this reason, it is still unclear how vitamin D relates to PPD.

To date, in the review by Amini et al., seven studies compared measured vitamin D levels during pregnancy or 24 h postpartum and observed their increase over time to identify whether serum 25-OHD levels could be linked to the risk of developing PPD. The results of most of these studies showed that lower serum 25-OHD levels were associated with the occurrence of PPD. However, no randomized, controlled trial was conducted [155].

### 7.5. Vitamin E

Vitamin E is a non-essential antioxidant. It reduces oxidative changes that result from stress. Low levels of antioxidants, which include vitamins C and E, have been linked to depression [159] as a result of reduced anti-radical activity. There is evidence to suggest that vitamin E supplementation positively affects mood changes in adults at risk of depression or with clinically diagnosed depression [160,161]. The metanalysis by Lee et al. indicates that the findings are contradictory, with some studies showing a significant reduction in symptoms of depression and anxiety, and other studies discovering no such relationship [45].

### 7.6. Zinc

Zinc (Zn) influences the regulation of various cellular processes as a cofactor of many enzymes [162]. Although the exact mechanisms of action are still unknown, it can be speculated that Zn has immunomodulatory properties and affects the neurological system [163]. The daily requirement for Zn during pregnancy is increased due to fetal and placental development [164]. Low serum Zn levels are a hallmark of depression and treatment-resistant depression [165].

Postnatal Zn supplementation in the mother may reduce the risk of developing postnatal depression [166].

### 7.7. Iron

Iron (Fe) is a component of hemoglobin in red blood cells (RBCs). Pregnant women are at risk of iron deficiency anemia due to higher maternal and fetal requirements, blood loss during pregnancy and childbirth, and significantly increased blood volume during pregnancy. Corwin et al. showed that women with iron-deficiency-induced anemia have an increased risk of PPD, but the pathogenesis of this phenomenon is still unknown [95]. On other hand, Ezzeddin et al. found that women who received iron supplements during pregnancy did not have reduced symptoms of PPD [167]. 

Nevertheless, another study found that iron supplementation during the postpartum period reduced the risk of PPD [136].

### 7.8. Selenium

Selenium (Se) acts as an anti-inflammatory component and a key antioxidant. According to Wang et al., selenium significantly modifies dopaminergic, serotonergic, and noradrenergic systems [168]. An increase in selenium levels significantly reduces PPD symptoms [169]. It was also shown that women receiving selenium supplementation have lower rates of depression [136,168]. Supplementation should be proceeded with caution because harmful levels of selenium can be easily achieved.

### 7.9. Magnesium

Magnesium (Mg) may also have antidepressant effects [170]. Patients with treatment-resistant depression have lower magnesium levels in the central nervous system [171].

Miller et al. showed an association between magnesium supplementation and a reduction in short-term depressive symptoms in nonpregnant individuals. They also demonstrated that perinatal magnesium was associated with lower severity of depressive symptoms in the immediate postpartum period [172,173,174,175]. 

However, there are not enough data examining the role of magnesium in preventing postpartum depression. It is presumed to reduce postpartum depressive symptoms by acting on NMDA receptors [172].

### 7.10. Conclusions

Deficiencies of the above-mentioned elements appear to increase the risk of developing PPD (Table 3). Therefore, it is important to test their levels in pregnant women and supplement any deficit. Particularly noteworthy are the B vitamins, as well as vitamin D, fluctuations of which may be crucial for whole groups of disorders, aside from depression. Their determinations should be carried out to correct deficiencies both during pregnancy as well as postnatally.

## 8. Review of Metabolic Markers and Other Metabolic Substances

### 8.1. Insulin

Insulin stimulates the absorption of excess glucose from the blood by other tissues to store energy. Insulin resistance (IR) occurs when cells become insensitive to insulin, leading to a buildup of glucose and insulin in the blood. From the second trimester of pregnancy, insulin sensitivity gradually decreases as the levels of this hormone increase. These changes are a physiological adaptation of the mother’s body to ensure an adequate supply of carbohydrates for the rapidly growing fetus. After delivery, insulin levels return to pre-pregnancy levels [176].

IR is a risk factor for depression. Studies also suggest that drugs that increase insulin sensitivity may play an important role in the treatment of classic depression, especially in patients with confirmed insulin resistance [172,177,178,179]. After childbirth, the risk of developing type 2 diabetes increases [180].

In addition, pre-gestational or gestational diabetes was independently associated with perinatal depression, including new incidences of postpartum depression [181].

There are also reports of insulin-like growth factor-1 (IGF-1) as perhaps an important marker of PPD. Women with higher serum levels of this factor in the first trimester of pregnancy were less likely to show postpartum depressive symptoms compared to those who had less [182].

### 8.2. Uric Acid

A decrease in serum uric acid levels has been observed in non-pregnant patients with depressive disorders compared to healthy subjects. These levels increased with the introduction of antidepressant pharmacotherapy [140].

Uric acid may also be a useful biomarker for differentiating depressive episodes in the context of MDD from bipolar affective disorder [59].

Further studies are needed to assess the diagnostic value of this marker in PDD as well.

### 8.3. Homocysteine

Homocysteine (Hct) is a substance formed in the metabolism of methionine which is an essential amino acid obtained from the daily diet [183]. 

Murphy et al. show that total maternal homocysteine level is significantly reduced in the first two trimesters of pregnancy compared to pre-conception concentrations [184].

A study by Zhang et al. using the LC-Q-TOF-MS method found significantly elevated urinary homocysteine levels in patients with postpartum depression [185].

Reducing homocysteine metabolism can inhibit the methylation reaction in the body, which affects phospholipid composition, cell membrane structure, and receptor function. Inhibition of myelin basic protein methylation can lead to looser myelin packing and reduced nerve conduction function. Homocysteine has also been shown to interact with transition metals, demonstrating a capacity for oxidative toxicity and nerve cell damage [186].

### 8.4. Tyrosine

Tyrosine is a precursor of brain catecholamines. 

In Zhang et al.’s study using the LC-Q-TOF-MS method, tyrosine levels were reduced in the urine of patients with postpartum depression, similarly to previous studies that found decreased levels in the blood of depressed patients [185], supporting that dopaminergic factors are involved in the development of clinical depression [187].

The Doornbos et al. study found increased levels of MHPG (3-methoxy-4-hydroxyphenylglycol), a metabolite of norepinephrine, in the cerebrospinal fluid of pregnant women experiencing postpartum depressive symptoms [188].

### 8.5. Vanillylmandelic Acid

Vanillylmandelic acid is a major metabolic product of norepinephrine, which plays an important role in the noradrenergic system and in the functioning of the hypothalamic-pituitary-adrenal axis. Its elevated levels are associated with generalized anxiety disorders [189]. 

Increased levels of this acid were found in the urine of patients with depressive symptoms during pregnancy [186].

A similar pattern was shown in other patients suffering from depression [190].

### 8.6. Alanine

Alanine is an essential amino acid that, among other things, acts as an inhibitory neurotransmitter in the brain. Its high levels in the early stages of pregnancy are considered a predictive marker of gestational diabetes [191].

In the context of postpartum depression, significantly higher urinary alanine levels were found in patients with depressive symptoms compared to controls [185].

These findings are consistent with observations of other depressed patients [140].

### 8.7. Conclusions

Further research is needed on metabolic markers of postpartum depression determined in a urine sample, such as alanine, vanillylmandelic acid, or homocysteine, among others, due to their minimal invasiveness and safety (Table 4).

## 9. Metabolic Lipid Markers

In psychiatric research, including PPD studies, the role played by lipids cannot be overlooked. Phospholipids account for 60% of brain dry weight [192].

The increase in lipid levels during pregnancy serves as an energy reserve to meet the metabolic needs of both the mother and the fetus, and in late pregnancy, lipid levels play an important role in milk formation before delivery. Toward the end of pregnancy, there is a significant decrease in fat deposition. The changes occurring in maternal lipid metabolism during pregnancy are associated with gestational age. Serum triglyceride (TG) and total cholesterol (TC) levels have been found to increase in pregnant women as gestational age progresses [193]. Previous studies have shown higher levels of total cholesterol, low-density lipoprotein cholesterol (LDL-c), and high-density lipoprotein cholesterol (HDL-c), which remain elevated in the postpartum period compared to the pre-pregnancy period [194].

Although a correlation between serum lipid levels and the development of depression has been demonstrated, there are still insufficient data on the relationship between lipid profile abnormalities and PPD. When assessing the relevance of lipids for the likelihood of depressive disorders in pregnant patients, it is important to consider the entire lipid profile rather than individual types of lipids, since so many have been linked to depression [195]. Among metabolic indicators, polyunsaturated fatty acid (PUFA) irregularities and changes in cholesterol levels appear to have the greatest potential as indicators of depression [18].

### 9.1. PUFAs

There are two main types of PUFAs: omega-3 and omega-6. Both are present in the brain, but each acts differently. While omega-6-PUFAs (e.g., arachidonic acid) are pro-inflammatory, omega-3-PUFAs (e.g., eicosapentaenoic acid, EPA) have anti-inflammatory properties [18].

In depression, abnormal blood levels of PUFAs are found, with decreased levels of eicosapentaenoic acid and other omega-3-PUFAs and increased levels of omega-6-PUFAs, including arachidonic acid, as evidenced by numerous studies [192,196,197].

Maternal PUFA concentrations during pregnancy are important for fetal lipid metabolism and adipocyte differentiation [198]. Hamazaki et al. found that women whose diets contained more n-3 PUFAs showed a reduced risk of postpartum depression 6 months after delivery and of serious psychiatric disorders 1 year after delivery [199]. 

Only a few studies focused on testing the validity of using n-3 PUFAs in the context of treating postpartum depression:

A treatment with different doses of a preparation containing docosahexaenoic acid (DHA) and eicosapentaenoic acid for 8 weeks reduced postpartum depression symptoms in a pilot study that did not include a placebo control group [200]. Study participants were randomly assigned to groups receiving: 0.5 g/day, 1.4 g/day, or 2.8 g/day of omega-3 fatty acids. Percentage reductions on the EPDS and HRSD scales were 51.5% and 48.8%, respectively, which can be considered significant values. In contrast, another study that administered DHA and EPA to patients for 8 weeks at a dose of 1.9 g/day or a placebo found no additional beneficial effect on postpartum depression when all the patients also received psychotherapy [201]. Similarly, a reduction in PPD symptoms was not observed in women who were given fish oil (EPA: DPA, 1.4: 1 from the 34th to 36th week of pregnancy until the 12th week postpartum) [202] and DHA supplements of 200 mg/day for 4 months postpartum or 220 mg/day from the 16th week of pregnancy until the 3rd month postpartum [200]. DHA dose may have been too low in this study to achieve optimal results, given the concentrations in the studies mentioned above.

### 9.2. Cholesterol

Cholesterol, next to PUFAs, is considered the most important biological lipid marker of depression. It does not pass through the blood–brain barrier, but is synthesized and recovered locally in the brain, mainly by oligodendrocytes [203].

Ramachandran et al. found that levels of total cholesterol (TC) and high-density lipoprotein cholesterol (HDL-c) were significantly lower in PPD women with severe depressive symptoms. The study found a significant negative correlation between HDL-c and EPDS score in women with PPD [204].

### 9.3. Conclusions

Given the importance of lipid markers in the development of inflammation and the increase in concentrations of individual components of the lipid profile with advancing gestational age and in the postpartum period, their collective determination may contribute to determining the risk of developing PPD in the context of inflammatory etiology. Among metabolic indicators of polyunsaturated fatty acid disorders, the above-discussed PUFA and changes in cholesterol levels seem to have the greatest potential as indicators of depression (Table 5).

## 10. Oxidative Stress in Pregnancy

In the first trimester of pregnancy, reactive oxygen species (ROS) play an important role in the adaptation of the endometrium and uterine muscle during embryo implantation. Pregnancy itself increases oxidative stress, which leads to an increase in circulating ROS. The placenta is considered the main source of ROS [205].

It is suspected that physiological hypoxia provides a kind of protection to the fetus against the teratogenic effects of ROS, which is most likely related to the multipotentiality of stem cells [206]. The partial pressure of oxygen inside the placenta (PO2) is two to three times lower between the 8th and 10th weeks of gestation (GW) compared to the 12th week of gestation. When the maternal blood circulation around the placenta is fully developed, there is a gradual increase in PO2 inside the placenta as the pregnancy progresses [207]. This happens when a balance is achieved between oxidative stress and the antioxidant capacity of the body [208]. Nevertheless, oxidative stress itself predisposes to the development of depression, so even physiological pregnancy can be considered a risk factor [209].

Among the factors associated with oxidative and nitrosative stress and the inflammatory theory of depression, the following are thought to be the most significant: MPO, NO and NOS, MnSOD, lipid peroxidase, and MDA [2].

### 10.1. MPO

Myeloperoxidase (MPO) is an inflammatory enzyme produced by activated leukocytes (nap). This compound of the hemoprotein group is accumulated inside the cells of the immune system—neutrophils, eosinophils, and monocytes [210]. MPO reacts with H_2_O_2_ (produced during an oxidative burst) and chloride ions to form hypochlorous acid/hypochlorite (HOCl/OCl-) [211]. HOCl then reacts with proteins to form HOCl-modified proteins. The presence of these chloramines is relevant to the development of inflammation. The presence of MPO in fetal membranes and basal lamina, as well as in maternal and fetal blood cells and placental tissues in humans, has been proven through immunoreactive method [212].

Monocytes/macrophages are the main cellular source of MPO in human placental tissues. Immunohistochemical studies have shown that HOCl modified proteins are present in the human placenta, but not in the first trimester of pregnancy (7th to 12th week) [212]. They have also been found in areas formed by cells of fetal origin and in maternal decidua tissues, i.e., where extranodal trophoblast cells infiltrate maternal tissue and stimulate the maternal immune system—therefore, the generation of modified HOCl proteins is considered physiological in pregnancy [213]. Nevertheless, it has been proven that HOCl protein levels are significantly elevated in MDD patients [214].

As has already been mentioned, MPO also accumulates in neutrophils, which differ in pregnancy from those in non-pregnant women since they cannot be fully activated to produce oxidative substances, but at the same time show higher levels of inactive forms of oxidative substances [212]. Immunofluorescence microscopy revealed the presence of MPO on the surface of neutrophils in pregnancy, while cells of non-pregnant women showed no MPO on the surface [213].

### 10.2. NO and NOS

Nitric oxide (NO) is a signaling molecule that plays a key role in the pathogenesis of inflammation. Under normal physiological conditions, it exerts anti-inflammatory effect. On the other hand, NO is considered a pro-inflammatory mediator that induces inflammation in pathological situations (it is then produced in increased amounts). NO is synthesized and released into endothelial cells with the help of NOS (nitrite synthases), which convert arginine into citrulline, thus producing NO [215]. Nitric oxide synthase (NOS) exists in two isoforms: neuronal NOS (nNOS), primarily involved in neurotransmission, and cytokine-induced NOS (iNOS). During pregnancy, vascular production of nitric oxide (NO) increases in the general body system and primarily in the uterine vasculature, which promotes stimulation of uterine blood supply [216].

The action of NO also leads to the vasodilation of the placenta. Increased iNOS production is also caused by progesterone [217].

NO levels are higher in patients with MDD [218], which may make them good markers of all depressive conditions including PPD.

### 10.3. MnSOD

Manganese superoxide dismutase (MnSOD) is an enzyme present in mitochondria. It is one of the first in the chain of enzymes involved in converting ROS formed by partial reduction of O2 [219].

MnSOD levels in the placenta during pregnancy affect fetal growth by reducing oxidative stress [217].

It is known that superoxide dismutase levels are reduced in placental trophoblast [220]. A number of studies have shown that SOD is altered in depression, but the results are not consistent. Most trials suggest that SOD activity is increased in depression [221,222,223], but contradictory results have also been reported [224,225]. A meta-analysis by Jimenes-Fernandes et al. found higher levels of SOD in MDD patients compared to healthy subjects [226]. However, studies indicating the relevance of SOD in the prediction of PPD are lacking.

MnSOD can be measured in maternal urine to show the relationship between its levels and the degree of lipid peroxidation [217]. 

### 10.4. Lipid Peroxidase

Lipid peroxidase is an enzyme that neutralizes free radicals formed by lipid peroxidation, i.e., as a result of the ROS/RNS action on lipids (e.g., cell membrane lipids). This process can self-perpetuate, leading to a cascade of lipid oxidation. The level of lipid peroxidase is an indicator of total lipid peroxidation in the serum, giving information about overall free radical activity in the body. Increased lipid peroxidation leads to antioxidant consumption [220]. The enzyme plays a very important role during pregnancy. Free-radical lipid peroxidation in human placental tissue microsomes increases with gestational age when a constant concentration of peroxidation catalysts (reduced nicotinamide adenine dinucleotide, adenosine diphosphate and ferric chloride) is supplied [208].

Lipid peroxidation is more marked in MDD patients than in control subjects [218]. A meta-analysis by Mazereeuw et al. [227] found a correlation between lipid peroxidation and the severity of depression. Physiological pregnancy increases lipid peroxidase levels [228], which may translate into an increased risk of depression.

Peripheral indicators of lipid peroxidation are good indirect markers for central nervous system concentrations [18].

### 10.5. Malondialdehyde (MDA)

MDA is one of the ultimate products of peroxidation of polyunsaturated fatty acids in cells [229]. An increase in free radicals contributes to its rise, so it can be considered a marker of oxidative stress.

At the end of pregnancy, attenuated natural IgM responses to oxidative specific epitopes (OSEs), including MDA, were found, indicating suppression of this part of the adaptive anti-inflammatory reflex of the immune system, which reduces excessive inflammatory responses triggered by pathogens or other immune stimulation [230].

These findings provide preliminary evidence that natural autoimmune responses to MDA exert a protective effect, inhibiting oxidative and nitrosative stress (O&NS) pathways through immune suppression and anti-tumor effects. Moreover, reduced natural autoimmune responses at the end of pregnancy may reflect impaired regulatory responses, contributing to increased O&NS pathways and a potentially increased risk of autoimmunity during pregnancy [231].

MDD patients have elevated MDA levels in the plasma, serum, and erythrocytes [232], although some studies do not support these findings [233,234,235]. The role of MDA in the development of PPD is not well understood; in view of the scarcity of information and the increased importance of MDA in pregnancy as well as its association with MDD, it is worthwhile to determine it peripherally in patients at risk of PPD.

### 10.6. Conclusions

Considering that pregnancy is associated with increased oxidative stress, which is considered a factor in the development of postpartum depression, peripheral determination of the above-mentioned enzymes may play an important role in identifying the risk of the onset of the disorder (Table 6). The minimally invasive testing methods may allow isolation of patients at risk for PPD and early implementation of therapy, especially since peripheral markers are proving to be good indirect markers for central nervous system concentrations. The most significant prognostic indicators appear to be MPO and lipid peroxidase.

## 11. Genetic and Epigenetic Factors

MDD has also been linked to genetic factors, most importantly those related to monoaminergic and glutamatergic signaling pathways [236]. Certain genes have been identified whose polymorphisms may be relevant to the development of MDD. Foremost among these are as follows: the gene encoding 5-HT transporter, 5-HT2A receptor, BDNF, TRP hydroxylase, SOD, and CAT [18]. It should, however, be mentioned that some studies contradict the existence of this relationship (despite the large group of study participants) [237]. A likely explanation for this discrepancy may be the significant heterogeneity of depression. Studies conducted by Couto et al. seem to confirm that genes contributing to classic depression are also present in PPD [238]. 

Epigenetic processes, mainly DNA methylation and histone modification, are involved in the development of depressive disorders, including PPD [18]. It is established that stress, whether physical or psychological, triggers these processes, leading to a heightened susceptibility to depression. Patients struggling with the disease show a decreased expression of BDNF and AcH3K14 genes and an increased expression of histone deacetylases in the hippocampus [239], as well as elevated levels of methylation of the promoter region of exon 1 in the BDNF gene compared to controls [240].

The 5HTT and COMT genes appear to be the most studied ones, with COMT-Val158Met significantly associated with PPD symptoms at 6 weeks, but not at 6 months postpartum [241]. Moreover, in a multivariate gene-environment model, COMT-Val158Met and maternity stressors correlated with PPD symptoms [242]. Among patients with a history of psychiatric problems, COMT-Val158Met and 5HTT-LPR risk variants were also linked to PPD symptoms [238].

The interaction effect between monoaminergic genes and environmental stressors is thought to be of great relevance to PPD susceptibility and have a possible predictive value [241]. Interestingly, the monoamine oxidase (MAO) gene in combination with COMT appears to regulate not only the response to stress in laboratory experiments, but also the stresses experienced during the perinatal period. Thus, the mental state of women during that time is most likely a product of the interplay of these polymorphic genes [243].

## 12. Limitations

The study is not a systematic review and does not provide quantitative information. Strict inclusion and exclusion criteria were not applied. Both large and small studies were included.

## 13. Conclusions

Existing scientific reports assessing the role of biomarkers in depression in the unique context of pregnancy are inconclusive, probably due to the dispersion of data between depressive subtypes, as well as the nature of changes occurring during pregnancy and childbirth. Consequently, biomarker determination in PPD carries some limitations. According to the authors of this paper, biomarkers are best studied collectively, correcting the analysis in view of individual patient characteristics; samples should be collected from blood or saliva after taking a history suggesting an endogenous cause of the disorder. The primary goal should be to discover the pathogenesis and determine the likelihood of PPD, taking into account the biological changes associated with pregnancy and childbirth, as well as the complexity of the disease itself. The most important diagnostic challenge remains in replacing complex and expensive analytical techniques with cheaper and readily available ones that can be applied to pregnant women. The most valuable goal of finding biomarkers of PDD is to prevent it by starting suitable treatment during pregnancy.

In our review, by far the most promising markers appear to be IL-10, IL-6, allopregnanolone, group D vitamins, MPO, lipid peroxidase, and lipid profile. It should be remembered that some of these substances during pregnancy and postnatally are (according to physiology) outside the general population norm values. In addition, the perinatal period itself, during which substances typically associated with high stress—e.g., TNF-α and others—may be a good marker of how significant a stressor childbirth itself might be, is regarded in this case as a trigger for depression (Table 7).

## Figures and Tables

**Table 1:** Hormones and their possible concentration in PPD.

Hormone		Concentration in PPD
estrogen		↓
progesterone		
allopregnanolone		
prolactin		↓
oxytocin		↑/↓
TSH		↓/N/↑
HPA	CRH	↓
	ACTH	
	cortisol	↓/↑

**Table 2:** Biomarkers of inflammation in PPD: possible concentrations.

Inflammation Biomarker		Concentration in PPD
Chemokines	CX3CL1	↓
	CXCL8	↑
	CCL2	↑
	CCR2	↓
	CCL4	↑
	CCL5	↑
	CCL11	↑
TNF-α		↑
TGF-β1		↓
TGF-β2		↑
TGF-β3		↓
INF-γ		↓
Interleukins	IL-1β	↑
	IL-2 i IL-2R	↑
	IL-3	↑
	IL-4	↓
	IL-6	↑
	IL-8	↑
	IL-10	↓
	IL-17	↑
	IL-18	↑
	BDNF	↓
CRP		↑

**Table 3:** Nutrients and their possible concentration in PPD.

Nutrient		Concentration in PPD
Vitamin A		↓
Vitamin B6		
Vitamin B9		
Vitamin B12		
Vitamin C		
Vitamin D		
Vitamin E		
Elements	Zinc	↓
	Iron	
	Selenium	
	Magnesium	

**Table 4:** Metabolic markers and other metabolic substances and their possible concentrations in PPD.

Metabolic Markers and Other Metabolic Substances	Concentration in PPD
Insulin	↓
Uric acid	↓
Homocysteine	↓
Tyrosine	↓
Vanillin-malic acid	↓
Alanine	↓

**Table 5:** Lipid markers and their possible concentrations in PPD.

Lipid Marker	Concentration in PPD
PUFA	↓
HDL-c	↓
cholesterol—total	↓

**Table 6:** Enzymes and their possible concentrations in PPD.

Enzyme	Concentration in PPD
MPO	↑
NO and NOS	↑
MnSOD	↑
lipid peroxidase	↑
MDA	↑

**Table 7:** Comparison of concentrations between selected biomarkers in physiological pregnancy and childbirth with PPD. The dash “-’’ separates the period of pregnancy from childbirth. N—normal, ↑—concentration increase, ↓—concentration decrease (values compared to nonpregnant women). The number of arrows highlights the intensity of the feature.

	Substance	Pregnancy—Postpartum Concentration	Possible Concentrations in PPD
hormones	estrogen	↑↑↑-N/↓	N/↓↓
	progesterone	↑↑-N/↓	N/↓↓
	allopregnanolone	↑↑-↓	↓↓
	cortisol	↑-N/↑	↑/N/↓
inflammatory markers	IL-1β	↑/↓-N	↑↑↑
	IL-6	↑/↓-N	↑↑↑
	IL-10	↑-↓	↓↓ ^1^
	TNF-α	↑/↓-N	↑↑
	BDNF	↓/N-↑	↓↓
	CCL-2	↑-N	↑↑
lipid markers	PUFA	↑-↓	↓↓
	cholesterol	↑-↑	↓↓
oxidative stress enzymes	MPO	↑-↓	↑↑
	lipid peroxidase	↑-↓	↑↑
nutrients	B vitamins	↓-↓/N	↓↓
	vitamin D	↓-↓/N	↓↓
metabolic markers	alanine	N	↑↑
	vanillylmandelic acid	N	↑↑
	homocysteine	N	↑↑

^1^ Predictive marker in pregnancy at 3rd trimester.
